# Induction and application of ferroptosis in cancer therapy

**DOI:** 10.1186/s12935-021-02366-0

**Published:** 2022-01-07

**Authors:** Qing Nie, Yue Hu, Xiao Yu, Xiao Li, Xuedong Fang

**Affiliations:** 1 China-Japan Union Hospital of Jilin University, Changchun, Jilin People’s Republic of China; 2 First Affiliated Hospital of Jilin University, Changchun, Jilin People’s Republic of China

**Keywords:** Ferroptosis, Mechanism, Inducers, Cancer therapy

## Abstract

At present, more than one cell death pathways have been found, one of which is ferroptosis. Ferroptosis was discovered in 2012 and described as an iron-dependent and lipid peroxidation-driven regulated cell death pathway. In the past few years, ferroptosis has been shown to induce tumor cell death, providing new ideas for tumor treatment. In this article, we summarize the latest advances in ferroptosis-induced tumor therapy at the intersection of tumor biology, molecular biology, redox biology, and materials chemistry. First, we state the characteristics of ferroptosis in cells, then introduce the key molecular mechanism of ferroptosis, and describes the relationship between ferroptosis and oxidative stress signaling pathways. Finally, we focused on several types of ferroptosis inducers discovered by scholars, and the application of ferroptosis in systemic chemotherapy, radiotherapy, immunotherapy and nanomedicine, in the hope that ferroptosis can exert its potential in the treatment of tumors.

## Introduction

**Fig. 1:**
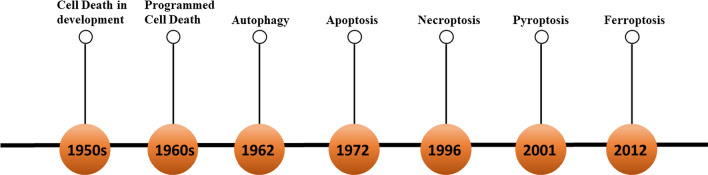
The progression of cell death

Cancer is the second leading cause of death in the world, causing approximately 10 million deaths each year. The treatment of cancer is currently one of the most researched topics. Eliminating cancer cells in the human body without affecting other healthy cells is the main concept of cancer treatment. Since the discovery of regulated cell death in the 1960s, people have realized that cell death is controllable and diverse (Fig. 1) [1]. RCD refers to the regulation of a series of specialized molecular mechanisms in pharmacology, molecular biology and genetics in the process of cell death [2, 3]. Caspase-dependent apoptosis has long been considered the only form of RCD [4], making anticancer drugs induce apoptosis of cells as one of the most important methods to kill cancer cells. However, in recent years, it has been discovered that cancer cells are resistant to drugs and have certain resistance to apoptosis [5, 6]. Therefore, targeting other forms of non-apoptotic cell death has become a new treatment approach to eliminate cancer cells and reduce the drug resistance of cancer cells.

In this series of non-apoptotic forms of RCD, ferroptosis was discovered in 2012 and described as a lipid peroxidation driven and iron-dependent RCD [7]. Although the term "ferroptosis" is a compound created after screening of small molecules that can inhibit the growth of RAS mutant cancer cells, in the past few years, ferroptosis has been shown to be closely related to the death of cancer cells [7, 8]. For example, the most classic p53 cancer suppressor gene can inhibit the expression of cystine/glutamate antiporter, thereby regulating ferroptosis [9]. In particular, cancer cells that are resistant to conventional therapies or have a high tendency to metastasize may be particularly susceptible to ferroptosis [10]. In addition, ferroptosis has recently been shown to be related to cancer immunotherapy, in which T cells and INFγ promote the sensitivity of cancer cells to ferroptosis [11]. In recent years, with the development of nanotechnology, the application of nanomedicine in cancer treatment has increased accordingly. Due to the unique physicochemical (high targeting efficiency, strong water solubility, low side effects) and some special properties (e.g., magnetic property, photothermal effect, electrochemical property, etc.), nanomaterials can kill cancer cells efficiently. And it is also found that nanomaterials can induce ferroptosis [12]. In this review, we first introduce the regulatory mechanism of ferroptosis and various death inducers, then elaborate on the current application status and possibilities of ferroptosis in cancer treatments, and finally express the expectations for its potential for clinical transformation.

## The basic characteristics of ferroptosis

The occurrence of ferroptosis is always accompanied by a series of variations in cellular, molecular, and genetic levels, which shares similarities and differences with other cell death modalities. Therefore, it is necessary to summarize the characteristics of ferroptosis and distinguish it from other cell death phenotypes. The confirmation of the ferroptosis phenotype mainly depends on the morphological changes at the cellular and subcellular levels and the expression of intracellular ferroptosis-related molecules (such as labile iron, ROS, peroxidized lipids and GSH). In addition, a series of related proteins and genes change when ferroptosis occurs [13]. Next, we will introduce a series of characteristics of morphology, molecular biology and genetics when ferroptosis occurs in cells.

### Morphological features

**Fig. 2:**
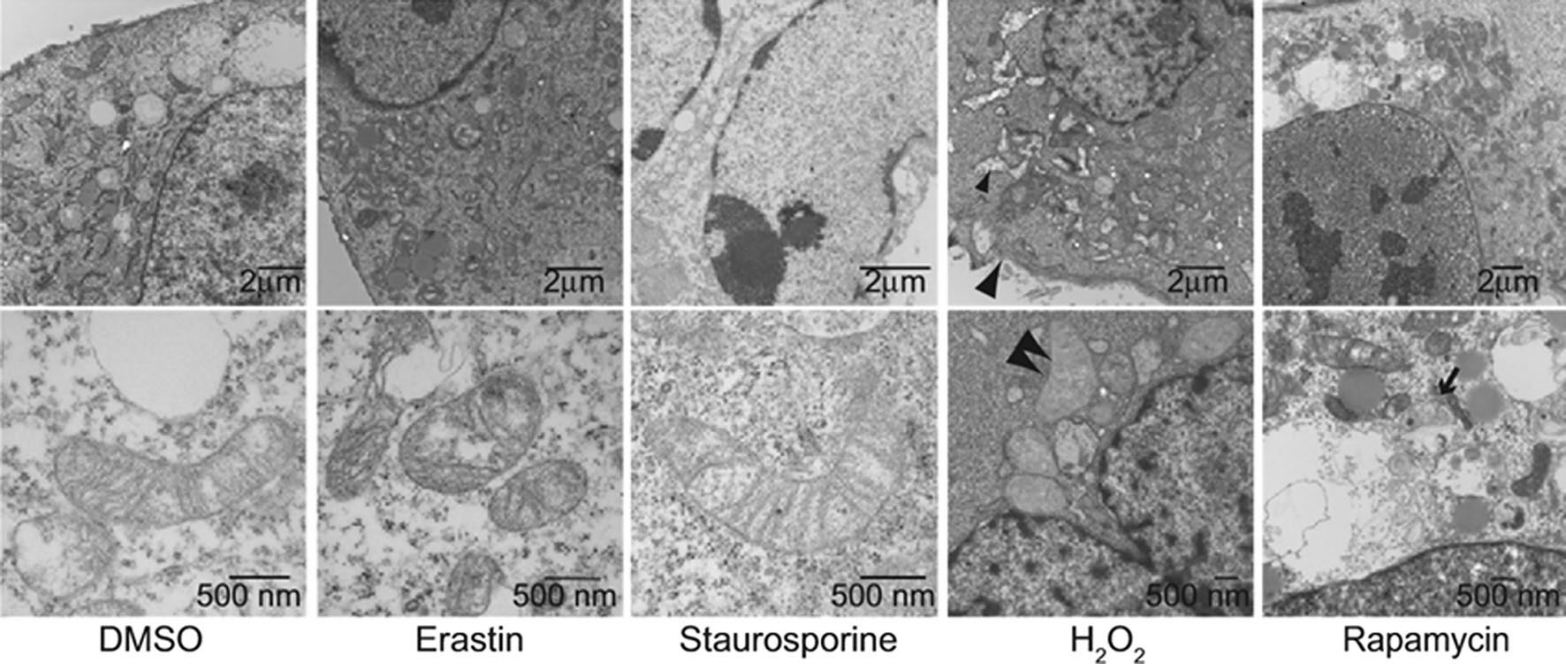
Morphological features. Transmission electron microscopy of BJeLR cells treated with DMSO (10 h), erastin (37 mM, 10 h), staurosporine (STS, 0.75 mM, 8 h), H_2_O_2_ (16 mM, 1 h), and rapamycin (Rap, 100 nM, 24 h). Single white arrowheads, shrunken mitochondria; paired white arrowheads, chromatin condensation; black arrowheads, cytoplasmic and organelle swelling and plasma membrane rupture; black arrow, formation of double-membrane vesicles. A minimum of 10 cells per treatment condition were examined

The iron-dead cells show morphological changes at the cellular and ultra-micro level: on the one hand, they lose the integrity of the plasma membrane, the cytoplasm is swollen (oncosis), the mitochondria are smaller than the normal cells, the mitochondrial cristaes shrink or disappear, the outer mitochondrial membrane ruptures, and the membrane density increases. On the other hand, the nuclei in ferroptotic cells remain structural integrity, without condensation or chromatin margination [7, 13]. In some special cases, ferroptosis is also accompanied by the detachment and aggregation of cells, as well as the increase of autophagosomes [14]. It is worth noting that ferroptosis occurs in one cell can quickly spread to neighboring cells [15, 16]. Whereas, apoptotic cells showed cell shrinkage and blebbing, fragmentation and marginalization of chromatin, accompanied by plasma membrane blebbing and the production of apoptotic bodies (Fig. 2). Apoptosis regulators (e.g., BCL-2 family members BAX and BAK) do not affect mitochondrial permeability [7, 17]. H_2_O_2_-induced necrosis is characterized by rupture of the plasma membrane and swelling of the cytoplasm and organelles, resulting in broken plasma membrane fragments, which are released and cause cell swelling [7, 18]. Autophagy induced by rapamycin always forms double-membrane enclosed vesicles [13]. However, these morphological features are not observed in ferroptotic cells (Fig. 2).

### Biochemical features

#### Iron accumulation

**Fig. 3:**
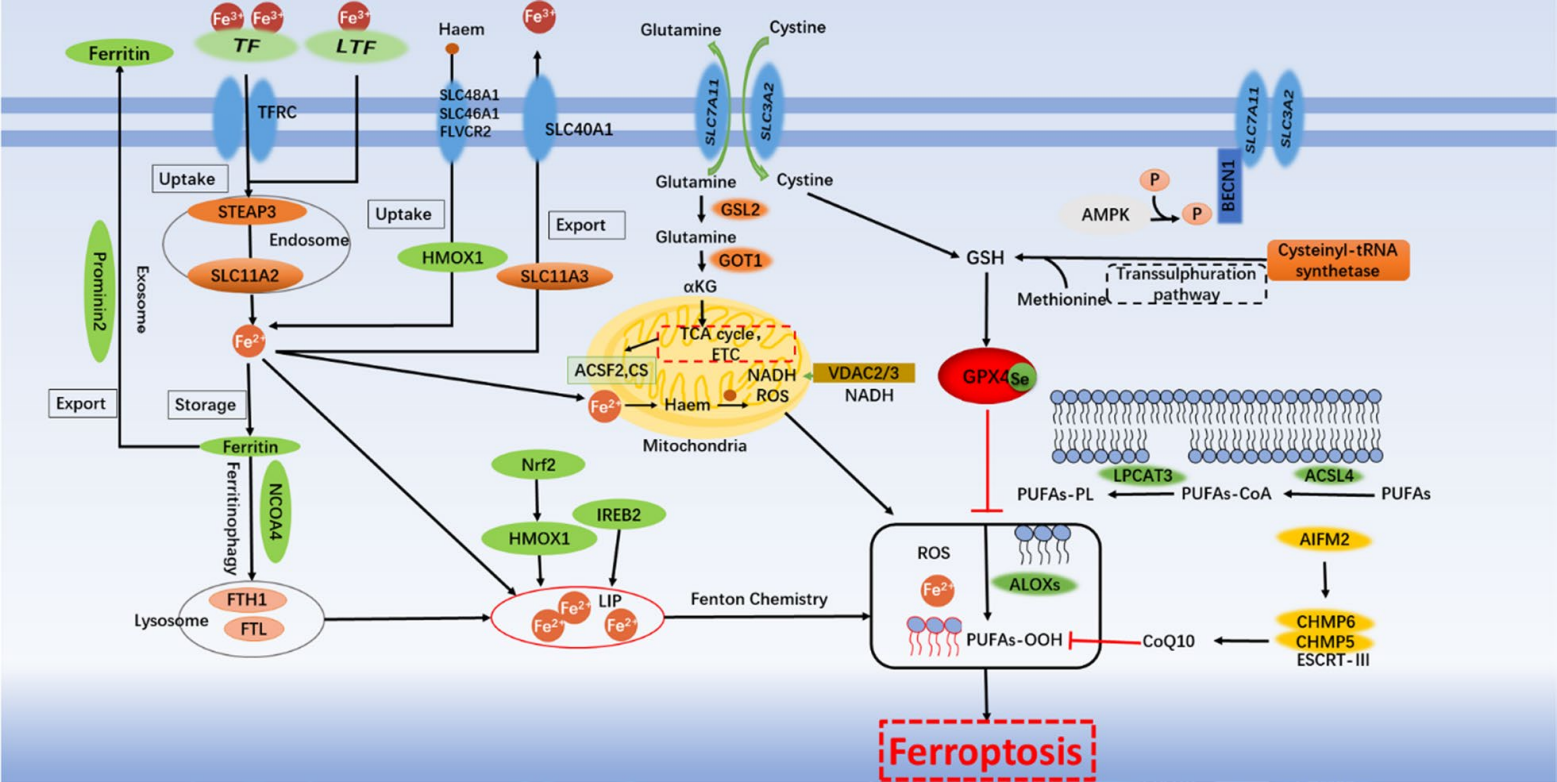
The regulatory mechanisms of ferroptosis

Compared with non-malignant cells, tumor cells have a stronger demand for iron. Researchers have increased the accumulation of iron in cells by increasing the absorption of iron ions by cells, reducing intracellular binding iron, reducing iron outflow, and by using various iron death inducers [19]. Excessive iron in cells can directly generate reactive oxygen species (ROS) through the Fenton reaction; or activate iron-containing enzymes (such as lipoxygenase ALOX or prolyl hydroxylase EGLN) to promote lipid peroxidation [20, 21]. It has also been discovered that free iron ions can also generate ROS through mitochondria. (Fig. 3) By inhibiting iron-related genes such as transferrin or using the DFO, it can effectively reduce intracellular free iron and effectively inhibit ferroptosis. At present, in various experiments, it has been found that a variety of metal elements can cause Fenton reaction, but it is still unclear why only iron can generate ROS through Fenton reaction in cells, and induce ferroptosis of cells [7]**.**

#### Lipid peroxidation

Lipid peroxidation is a reaction driven by free radicals, which mainly affects the polyunsaturated fatty acids (PUFAs) in the cell membrane. PUFAs are most prone to peroxidation, which leads to the destruction of lipid bilayers and affects membrane function [22–24]. The products of lipid peroxidation include the initial lipid hydroperoxides (LOOHs) and subsequent reactive aldehydes (e.g., malondialdehyde (MDA) and 4-hydroxynonenal (4-HNE)), which will increase during ferroptosis. Different lipoxygenases, especially ALOXs, have an up-and-down related role in mediating lipid peroxidation to produce the hydroperoxides, thereby promoting ferroptosis [24, 25]. Various cell membrane lipids (e.g., phosphatidylcholine, phosphatidylethanolamine (PE) and cardiolipin) may be oxidized [25]. Several membrane electron transfer proteins, especially NADPH oxidase (NOX), contribute to the production of ROS for lipid peroxidation in ferroptosis. In other cases, mitochondria participate in the induction of ferroptosis through processes such as the electron transport chain, the tricarboxylic acid cycle, the breakdown of glutamine, and the synthesis of lipids. Although mitochondria undergo strong changes in the process of ferroptosis, cardiolipin peroxidation has not been found in ferroptosis, and the role of mitochondria themselves in the process of ferroptosis is also controversial [26–28]. In different types of cancer cells, the occurrence and development of lipid peroxidation may be different.

### Genetic features

As early as 2012, the ferroptosis activator erastin was identified because it can selectively trigger cell death in cancer cells harbouring mutant but not wild- type RAS. The cell death induced by erastin activates RAS-RAF-MEK-ERK pathway [29, 30]. Subsequent research identified some proteins and genes that can be considered as biomarkers of ferroptosis. It has recently been shown that KRAS is involved in the regulation of multiple metabolic pathways such as ROS production, glutamine metabolism and the TCA cycle, making tumor cells in a delicate high-energy state. In this state, once the oxidation-antioxidant balance is broken (excessive ROS production or reduced GSH), ferroptosis is prone to occur [31, 32]. Genes such as prostaglandin endoperoxide synthase 2 (PTGS2/COX2), Acyl-CoA synthase long-chain family member 4 (ACSL4), and nuclear factor erythrocyte-like 2 (NRF2/ NFE2L2) etc. PTGS2 does not use prostaglandins as a substrate for lipid peroxidation, but can oxidize lysophospholipids. PTGS2 is generally considered to be a biomarker of ferroptosis, but it is not a driving factor [33]. ACSL4 is involved in the synthesis of fatty acids and is considered to be a specific biomarker and driving factor of ferroptosis. (Fig. 3) [22, 25] The activation of NRF2 can inhibit the occurrence of ferroptosis, and excessive activation may promote ferroptosis. In addition, some traditional tumor suppressor genes have also been found to be related to ferroptosis [34]. For example, p53-mediated SLC7A11 transcriptional inhibition promotes ferroptosis in cancer cells. But p53-regulated ferroptosis does not depend on the GPX4-ACSL4 pathway, suggesting that may be other ways in the ferroptosis process regulated by p53 [9]. For example, p21 is responsible for encoding a 21kd protein of RAS family, it can prevent p53-induced ferroptosis by adjusting ROS levels [35]. Subsequent research identified a complex signaling pathway that regulates ferroptosis by generating excess ROS through iron accumulation and lipid peroxidation. This network is of special significance as a regulatory pathway for RCD in oncology.

## Regulation mechanism of ferroptosis

### Iron metabolism

The iron in the circulatory system is mainly Fe^3+^ [36], which is present in serotransferrin- mediated or lactotransferrin- mediated iron, and is combined with endocytosis through the cell membrane transferrin receptor 1 (TFR1) into the cell to form endosomes. In endosomes, the STEAP3 metalloreductase reduces Fe^3+^ to Fe^2+^, and then releases Fe^2+^ from endosomes into the cytoplasm through solute carrier family 11 member 2 (SLC11A2/DMT1), and excess iron is stored in ferritin [37]. In the case of balanced iron metabolism, very little iron enters and leaves the cell every day. When iron accumulates too much, a small iron pool (the labile iron pool, LIP) containing Fe^2+^ is formed in the cell, which can directly catalyze the formation of ROS through the Fenton reaction. In addition, iron and iron derivatives (heme or iron-sulfur [Fe-S] clusters) [38], which is vital to the activity of enzymes that can catalyze the production of ROS (eg., nicotinamide adenine dinucleotide phosphate hydride (NADPH) oxidases (NOXs), LOXs, and mitochondrial electron transport complexes), which can promote the production of ROS, and further promote lipid peroxidation induce ferroptosis [39]**.**

Non-canonical ferroptosis induction refers to it caused by increasing LIP, such as increased expression of TFR1, decreased expression of ferritin, decreased expression of iron transporter, or excessive activation of heme oxygenase 1 (HMOX1) [40]. In cells with mutations in the RAS gene, the expression of TFR1 increases, while the expression of ferritin, which stores iron, decreases, increasing the Fe^2+^ content of LIP in the cell [30, 41]. Iron response element binding protein 2 (IREB2) gene which is the main transcription factor gene silencing can increase the expression of ferritin heavy and light chains and reduce the iron content in cells, indicating that IREB2 can indirectly regulate iron in the cell [7, 39]. HMOX1 has antioxidant activity, and excessive activation of HMOX1 can catalyze the degradation of heme into ferrous iron, biliverdin and carbon monoxide, and the increase of Fe^2+^ can induce ferroptosis [34, 42]. When free Fe^2+^ increases, the oxidative stress in the mitochondria will also increase, and the production of ROS will increase, which promotes lipid peroxidation and induces ferroptosis [39] Iron chelating agents inhibit ferroptosis by limiting iron overload, and increasing exogenous iron can promote ferroptosis [43]. For example, using iron such as iron chloride, hemoglobin, hemin or ferrous ammonium sulfate to overload the LIP of cells, inducing the non-canonical ferroptosis. But what is reassuring is that LIP only accounts for a small part of the total iron of the cell. The vast majority of iron is stored in ferritin or used by the cell in the metalloprotein.

Excess iron in cells is stored in ferritin, which is composed of ferritin light chain (FTL) and ferritin heavy chain 1 (FTH1), which absorb iron ions in the form of multimers and store them in the cytoplasm. Among them, FTH1 contains a ferrous oxidase center, which can quickly convert Fe^2+^ to Fe^3+^ and store it in ferritin. Lysosomes degrade ferritin through nuclear receptor coactivator 4 (NCOA4) to increase free iron levels (this process is called ferritin autophagy), inhibit NCOA4-mediated ferritin autophagy, which can increase iron storage and limit ferroptosis happen [44–46]. Finally, solute carrier family 11 member 3 (SLC11A3, also known as ferroportin) can oxidize Fe^2+^ to Fe^3+^, and then iron-efflux protein solute carrier family 40 member 1 (SLC40A1/ferroportin1/FPN) squeezes Fe^3+^ into the extracellular space [37, 46]. Alternatively, prominin 2 (a member of the prominin family of pentaspan membrane glycoproteins, PROM2) exports iron by forming ferritin-containing exosomes in epithelial and breast cancer cells, leading to ferroptosis resistance [47]**.**

Epidemiological evidence suggests that high dietary iron intake increases the risk of several cancers, such as hepatocellular carcinoma (HCC) and breast cancer [2]. Through the understanding of iron metabolism, it is found that the changes in total iron levels in the human body are mainly caused by Fe^3+^; while the occurrence of ferroptosis is mainly induced by the increase of intracellular Fe^2+^. Increasing iron intake or reducing iron output will make cancer cells sensitive to oxidative damage and ferroptosis. Ferroptosis-mediated cancer targeted therapy has limitless prospect.

### Lipid peroxidation

During ferroptosis, polyunsaturated fatty acids (PUFAs), especially arachidonic acid (AA) and adrenic acid (AdA), are most prone to peroxidation to produce lipid hydroperoxides (LOOHs) and subsequent reactive aldehydes (such as 4-HNEs or MDAs) cause damage to the lipid bilayer and affect membrane function [22–25]. The biosynthesis and remodeling of PUFAs in cell membranes are mainly regulated by ACSL4 and lysophosphatidylcholine acyltransferase 3 (LPCAT3) [47]. ACSL4 catalyzes the combination of free AA or AdA and CoA to form derivatives AA-CoA or AdA-CoA, and LPCAT3 then promotes their esterification to membrane phosphatidylethanolamine to form AA-PE or AdA-PE. If ACSL4 is knocked down, AA will be converted into acylated AA; or LPCAT3 will be silenced, which catalyzes the insertion of acylated AA into PLs, making cells resistant to ferroptosis. The up-regulation of ACSL4 is considered to be a biomarker and contributor to ferroptosis [23, 48]. ACSL3 converts monounsaturated fatty acids (MUFAs) into acyl-CoA esters to incorporate membrane phospholipids, thereby protecting cells from ferroptosis [49]. At the same time, studies have found that peroxisome (PEX)-mediated plasmalogen biosynthesis provides another source of PUFA for lipid peroxidation during ferroptosis, indicating that PEX may promote lipid peroxidation related to ferroptosis [50]**.**

The peroxidation process is mainly divided into two categories: enzymatic lipid peroxidation and non-enzymatic lipid peroxidation [51]. Enzymatic lipid peroxidation is mainly mediated by the activity of the arachidonate lipoxygenase (ALOX) family in a controlled manner. The mammalian ALOX family consists of six members (ALOXE3, ALOX5, ALOX12, ALOX12B, ALOX15 and ALOX15B). Different lipoxygenases mediate lipid peroxidation to produce hydroperoxides(AA-PE-OOH or AdA-PE-OOH) has a context-dependent role to promote ferroptosis. For example, ALOX5, ALOXE3, ALOX15, and ALOX15B are responsible for ferroptosis in human cell lines derived from various cancer types (BJeLR, HT-1080 or PANC1 cells), while ALOX15 and ALOX12 mediate the derivation of non-small-cell lung cancer (NSCLC) p53 induced ferroptosis in H1299 cells, in which ALOX12 induced ferroptosis through TP53-mediated down-regulation of SLC7A11 [20, 24, 52, 53]. Current studies have found that inhibiting or knocking down lipoxygenase can inhibit ferroptosis in certain cell types. It is still unknown whether other oxygenases (such as cyclooxygenase and peroxygenase) also play a similar role in lipid peroxidation [33]**.**

Non-enzymatic lipid peroxidation is a free radical-driven chain reaction. Reactive oxygen species (ROS) initiate the oxidation of PUFAs, which mainly involve hydrogen reactions. In the presence of Fe^2+^, the Fenton reaction generates hydroxyl radicals (a highly mobile, water-soluble ROS that can trigger lipid peroxidation) [51]. The hydroxyl radicals extract hydrogen from PUFAs to form carbon-centric lipid radicals(L^.^) [54]. Molecular oxygen (O_2_) reacts quickly with lipid radicals to produce lipid peroxy radicals (LOO^.^). Subsequently, LOO^.^ acts as a catalyst to extract hydrogen from PUFAs to form lipid hydroperoxides (LOOH) and new LOO^.^. And LOOH can be converted into alkoxy radicals (LO^.^), which reacts with adjacent PUFAs to initiate another chain reaction. Lipophilic antioxidants can reduce ROS by reducing reactions or combine with peroxides generated by the auto-oxidation reaction by releasing hydrogen atoms, interrupting the chain reaction. Or iron chelating agents such as DFO, which can be complexed iron interrupts the reaction [51]**.**

Although the exact mechanism of lipid peroxidation leading to ferroptosis in cells is unknown, current studies have found that lipid peroxides have toxic effects on cancer cells through two mechanisms. Molecularly, lipid peroxides are further broken down into active substances, which can consume amino acids, nucleic acids and proteins, driving cells to ferroptotic death [54]. In addition, lipid hydroperoxides may be broken down into reactive toxic aldehydes, such as 4-HNEs or MDAs, which by crosslinking may inactivate proteins involved in essential cellular processes to promote ferroptosis [55]. Structurally, extensive lipid peroxidation leads to biofilm thinning and increasing curvature, leading to a vicious cycle of lipid peroxidation, and ultimately leading to membrane instability and the formation of lipid pores (similar to the proteinaceous pores observed in necroptosis and pyroptosis) and micelle formation [40, 56–59]. Or continuous large-scale oxidation and consumption of PUFAs may change the fluidity and structure of the membrane, and increase the permeability of the membrane, leading to the loss of membrane integrity, and ferroptosis of cancer cells [60]**.**

### Oxidation

ROS is a group of molecules containing partially reduced oxygen, which can cause cancer cells to die by destroying biological molecules such as DNA/RNA, proteins, and lipids [54]. ROS involved in ferroptosis can be produced from various sources, and the accumulation of oxidation products (especially phospholipid hydroperoxide) is considered to be a sign of ferroptosis [59]. ROS is a by-product of aerobic metabolism. In the presence of mitochondrial superoxide dismutase (SOD), the electron transport chain on the inner mitochondrial membrane produces H_2_O_2_, which then diffuses from the mitochondria to the cytoplasm. The rate depends on the mitochondrial transmembrane potential. At a high iron concentration that is conducive to the Fenton reaction, H_2_O_2_ forms highly reactive oxygen radicals to promote lipid peroxidation [28, 52, 61]. In order to deal with the excessive production of ROS, there is a complete anti-oxidative stress system in the cell.

#### Antioxidant mechanism

##### xCT

Amino acids cannot diffuse directly into cells, they must be transported across the cell membrane with the help of specific transport proteins. The amino acid antiporter system Xc^−^ is one of transporters and consists of two core components: the light chain SLC7A11 (xCT) and the heavy chain SLC3A2 (4F2hc). It introduces extracellular oxidized form of cysteine and cystine to exchange intracellular glutamate. After cystine enters the cell, it is reduced to cysteine, and cysteine ​​is involved in the synthesis of GSH (a major endogenous antioxidant) [7]. Under the conditions of extracellular oxidation, the exchange of cystine and glutamate is the most upstream event of ferroptosis. The inhibition of the SLC7A11 pathway is the most critical upstream mechanism for inducing ferroptosis [34, 62]. Its expression or activity is regulated by many factors. For example, under the positive regulation of Nrf2 and the negative regulation of cancer suppressor genes (such as TP53, BAP1 and BECN1) [63, 64], they form a complex network to control the level of GSH in ferroptosis. Small molecule compounds or drugs (eg., erastin, sorafenib and sulfasalazine) inhibit SLC7A11 or reduce glutamate and cause glutathione depletion to trigger ferroptosis [49, 65]. AMPK is the main regulator of ATP homeostasis (Fig. 2). Its mediated BECN1 phosphorylation promotes ferroptosis by inhibiting SLC7A11 activity, while mediated Acetyl CoA carboxylase(ACACA) phosphorylation inhibits ferroptosis by inhibiting fatty acid biosynthesis, indicating that the energy state may be affect the lipid biosynthesis and peroxidation during the ferroptosis [61, 66]. When the system Xc^−^ is inhibited, the trans-sulfuration pathway (methionine through which cysteine ​​is provided from cystathionine for glutathione synthesis) regulates ferroptosis in certain cells, which is regulated by the aminoacyl tRNA synthetase family (such as CARS1). Up-regulation of the transsulfur pathway can make cells insensitive to ferroptosis induced by erastin [67]. Recently, the deubiquitinating enzyme ubiquitin aldehyde binding 1 (OTUB1), a member of the ovarian cancer (OTU) family, has been identified as an important factor in stabilizing SLC7A11. The inactivation of OTUB1 makes cancer cells sensitive to ferroptosis. In addition, OTUB1 is overexpressed in cancer cells, making OTUB1 a potential target for ferroptosis-mediated cancer treatment [68]**.**

##### GSH

GSH is the main antioxidant in mammalian cells and can be used as a cofactor of selenium-dependent GPX4 to reduce lipid hydroperoxides [69]. Ferroptosis can be triggered by GSH consumption (such as the consumption of GSH by erastin indirectly inactivates GPX4) or direct reduction of GSH synthesis (such as the inhibition by buthionine sulfoximine (BSO)), which leads to ROS accumulation and subsequent lipid peroxidation [70, 71]. It should be noted that, researchers have found that lack of cystine can inhibit cell growth, while lipophilic antioxidants and iron chelators agents can inhibit this type of cell death [39]. Multidrug resistance protein 1 (MRP1) is an adenosine triphosphate (ATP) binding cassette family transporter that can export certain types of chemotherapeutic drugs. Cancer cells with high MRP1 expression show a multidrug resistance phenotype. Recently, MRP1 has been identified as a negative regulator of intracellular GSH levels, and high MRP1 expression can effectively sensitize cancer cells to ferroptosis inducers that target GSH metabolism [72]. The study not only provides a potential strategy to eradicate drug-resistant cancers, but also explains from another perspective why some aggressive malignancies are sensitive to ferroptosis (Fig. 3).

##### GPX4

The classic pathway induces ferroptosis through peroxidative damage to the membrane, and the main detoxification mechanism is achieved through the catalytic detoxification of GPX4. Early experiments found that mice that knock out or silence the GPX4 gene cannot survive, proving that GPX4 is an essential gene for survival. As a central downstream regulator of ferroptosis, GPX4 uses two molecules of GSH as electron donors to reduce toxic phospholipid hydroperoxides to non-toxic phospholipids, even if they has been inserted into the membrane or lipoproteins to combat lipid peroxidation [25, 33]. The relationship between GPX4 expression and patient survival outcome depends on the cancer type. For example, the high expression level of GPX4 is negatively correlated with the prognosis of breast cancer patients [73], but has good survival outcomes for pancreatic cancer patients [74]. This may be related to the KRAS mutation in patients with pancreatic cancer. In PDAC cells, mutant KRAS transcription activates NRF2, and NRF2 up-regulates xCT, thereby regulating GPX4 expression [32]. The expression and activity of GPX4 in ferroptosis depend on the presence of GSH and selenium. When synthesizing GPX4, the nascent polypeptide chain combines selenium into selenocysteine ​​(Sec), where selenium replaces the sulfur of cysteine, increasing the anti- ferroptosis activity of GPX4 [75, 76]. GPX4 can be inactivated through direct or indirect targeting mechanisms. Such as direct pharmacological (eg., RSL3, altretamine, ML162, ML210, FIN56 or FINO_2_) or genetic (Cre recombinase method) interventions to GPX4 can induce ferroptosis. In addition, consumption of GSH is an indirect method of inactivating GPX4 [77, 78]. GPX4 depletion also mediates other non-ferroptosis RCDs (e.g., apoptosis, necroptosis and pyroptosis) [78–80], indicating that lipid peroxidation is located at the crossroads of several of these pathways, but downstream effectors may be different. Although GPX4 inhibition is an important downstream signal in the process of ferroptosis, ferroptosis unrelated to GPX4 may still occur. For example, TP53-mediated ferroptosis does not need to inhibit GPX4, although TP53 can inhibit SLC7A11 expression [9]**.**

#### Other oxidative stress pathways

##### Nrf2

In addition to GPX4, antioxidant proteins such as Nrf2 are also the main regulators of oxidative stress signals, and can inhibit lipid peroxidation. However, excessive activation may induce ferroptosis through unstable iron-catalyzed ROS metabolism mediated by HMOX1 [81–83]. Nrf2 also has a dual role in cancer progression, lack of Nrf2 activity can contribute to early cancerigenesis, while high constitutive Nrf2 activity can trigger cancer progression and resistance to treatment [81]. Nrf2 activates the protective genes by transactivating iron metabolism, oxidative defense, and redox signaling [34, 84]. Preclinical studies have shown that Nrf2 signaling is an important defense mechanism against ferroptosis and is involved in the resistance of HCC cells to sorafenib. After erastin and sorafenib are used to inhibit or silence Nrf2 genes, the sensitivity of cells to ferroptosis increases, which emphasizes its key role in antioxidant mechanisms [34, 81, 85]. The contribution of Nrf2 to ferroptosis resistance and the therapeutic potential of Nrf2 inhibitors (such as brusatol and trigonelline) to enhance the treatment of ferroptosis need to be further addressed in preclinical and clinical studies.

##### HIF

Hypoxia-inducible factor (HIF) plays a central role in the response to oxidative stress, affecting various pathological conditions of tissues and cells, and is a key factor in intracellular metabolism. The main regulator of hypoxia HIF1 is a heterodimeric transcription factor, including an unstable α-subunit (including HIF1A, endothelial PAS domain protein 1 (EPAS1, also known as HIF2A) and HIF3A) and stable β subunit (for example, aromatic hydrocarbon receptor nuclear transport protein (ARNT1/HIF1B)). The expression of HIF1A and EPAS1 are elevated in a variety of cancer types, and is usually associated with a poor prognosis of patients [86, 87]. Under normoxic conditions, HIF1A and EPAS1 are hydroxylated by members of the EGLN family of hypoxia-inducible factors, and then recognized by the E3 ubiquitin ligase VHL for proteasomal degradation. Under hypoxic conditions, the inactivation of hydroxylase causes HIF1A and EPAS1 to accumulate and form heterodimers with ARNT, thereby inducing the transcription of genes involved in hypoxia adaptation and survival [87]. EGLN protein is not only an iron-dependent sensor of oxygen, but also an iron-dependent sensor of cysteine, used to catalyze the hydroxylation of HIF. EGLN is a key target of iron chelator (such as deferoxamine), which can increase the stability of HIF by inhibiting the activity of EGLN to prevent ferroptosis by ischemia–reperfusion injury [86]**.**

In early studies, HIF had a dual role in regulating the ferroptosis of cancer cells. In HT-1080 fibrosarcoma cells, hypoxia-induced HIF1A expression inhibits ferroptosis by increasing the expression of fatty acid-binding proteins 3 and 7 to promote fatty acid uptake and increase lipid storage capacity to avoid subsequent lipid peroxidation [88]. In renal cell carcinoma (RCC), hypoxia induces EPAS1 activation and up-regulates the expression of HILPDA to promote PUFAs production and subsequent lipid peroxidation, and induce ferroptosis [89, 90]. Therefore, effective control of HIF is necessary to maintain lipid homeostasis to regulate ferroptosis. In clinical trials, we can consider using HIF inhibitors to regulate ferroptosis according to the situation.

## Inducers of ferroptosis

Since ferroptosis was discovered, many researchers have tried to discover all the activation methods that induce ferroptosis, hoping to target the induction of ferroptosis in cells through different ways to achieve the purpose of suppressing cancers. At present, experiments have shown that various small molecule drugs can initiate ferroptosis in four ways: Class I ferroptosis inducers (FINs) mainly consume intracellular GSH, class II FINs mainly target GPX4 and inactivate its activity, class III FINs mainly consume GPX4 and endogenous antioxidant CoQ10 through the SQS-mevalonate pathway, class IV FINs induce lipid peroxidation by increasing the LIP or oxidizing iron [51]. There are still some small molecule compounds, due to their complex mechanism of action, cannot be specifically judged as one of the above four categories. Now summarized in other ferroptosis inducers (Table 1).

**Table 1:** Inducers of ferroptosis

Target	Compound/Drug	Mechanism	Tumour type	Refs
Class I FINs				
SLC7A11	Erastin	Inhibit SLC7A11 and prevent cystine import, combine with VDAC2/3	Glioma, lung cancer, fibrosarcoma, melanoma, breast cancer, cervical cancer, RCC	[7, 29, 30]
	Piperazine erastin (PE)	Inhibit SLC7A11 and prevent cystine import	Fibrosarcoma	[33]
	Imidazole ketone erastin (28)	Inhibit SLC7A11 and prevent cystine import	DLBCL	[20]
	Sulfasalazine	Inhibit SLC7A11	Breastcancer, glioblastoma, fibrosarcoma, NSCLC, prostate cancer	[7, 97]
	Sorafenib	Inhibit SLC7A11	AML, HCC, neuroblastoma, NSCLC, RCC	[65]
	Glutamate	Inhibit SLC7A11	-	[7, 17]
GCL	Buthionine sulfoximine (113)	Inhibit the GCL and reduce GSH synthesis	Melanoma, neuroblastoma	[33, 70, 115]
GSH	Cyst(e)inase	Degrade cysteine and cystine, reduce GSH levels	Prostate cancer, chronic lymphocytic leukemia and pancreatic cancer	[107, 116]
	Cisplatin	Combine with GSH to inactivate GPX4	Ovarian cancer, pancreatic cancer, NSCLC, urothelial cancer	[94, 117–119]
Class II and III FINs				
GPX4	RSL3	Inhibit GPX4 directly	Fibrosarcoma, NSCLC, pancreatic cancer, leukemia	[19, 33, 120]
	FIN56	Combine and activate SQS to reduce CoQ10	Fibrosarcoma	[77, 111]
	ML162(DPI7), DPI12, ML210(DPI10), DPI13	Inhibit GPX4 covalently	-	[33]
	Altretamine	Inhibit GPX4	Lymphoma, sarcoma, ovarian cancer	[77]
	FINO_2_	Oxidize Fe^2+^ and PUFAs, promote the accumulation of ROS; indirectly inactivate GPX4;	Fibrosarcoma	[33, 55]
Class IV FINs				
Iron	Heme	Up-regulate HMOX1 expression and increase LIP	Glioblastoma, leukemia	[34, 38]
	Withaferin A	Up-regulate HMOX1 expression and increase LIP at middle dose and inactivate GPX4 at high doses	Breast cancer, Neuroblastoma	[82, 121]
	BAY 11–7085	Up-regulate HMOX1 expression and increase LIP	CRC, cervical cancer	[83]
	Artesunate	Oxidize Fe^2+^, promote the accumulation of ROS, induce ferritinophagy	Pancreatic cancer	[122–126]
	Dihydroartemisinin	Oxidize Fe^2+^, promote the accumulation of ROS, induce ferritinophagy and inhibit ferritin synthesis	Ovarian cancer	
	Siramesine, lapatinib	Decrease SLC40A1, increase transferrin and LIP	Breast cancer	[113]
	Neratinib	Decrease SLC40A1, increase transferrin and LIP	Breast cancer, CRC	[127]
	Salinomycin	Decrease SLC40A1, increase transferrin and LIP	Various solid tumour types	[128]
Others				
ROS	BAY 87–2243	Combine with mitochondrial respiratory chain complex I	NSCLC	[83]
FSP1	iFSP1	Inhibit the reduction of CoQ10 by FSP1	Fibrosarcoma, NSCLC	[109, 110]
HMGCR	Statins	Combine as lipid- lowering agent, in oncology phase I trials; CoQ10 deletion	Breast cancer, AML, HCC, MM, Fibrosarcoma, NSCLC	[111, 127, 129]
Nrf2	Trigonelline, brusatol	Nrf2 inhibition	HCC, NSCLC	[34, 130]

*AML* acute myeloid leukaemia, *CRC* colorectal cancer, *GSH* glutathione; *HCC* hepatocellular carcinoma, *MM* multiple myeloma, *NA* not available, *NSCLC* non- small- cell lung cancer, *RCC* renal cell carcinoma, *DLBCL *diffuse large B cell lymphoma

### Class I FINs

**Fig. 4:**
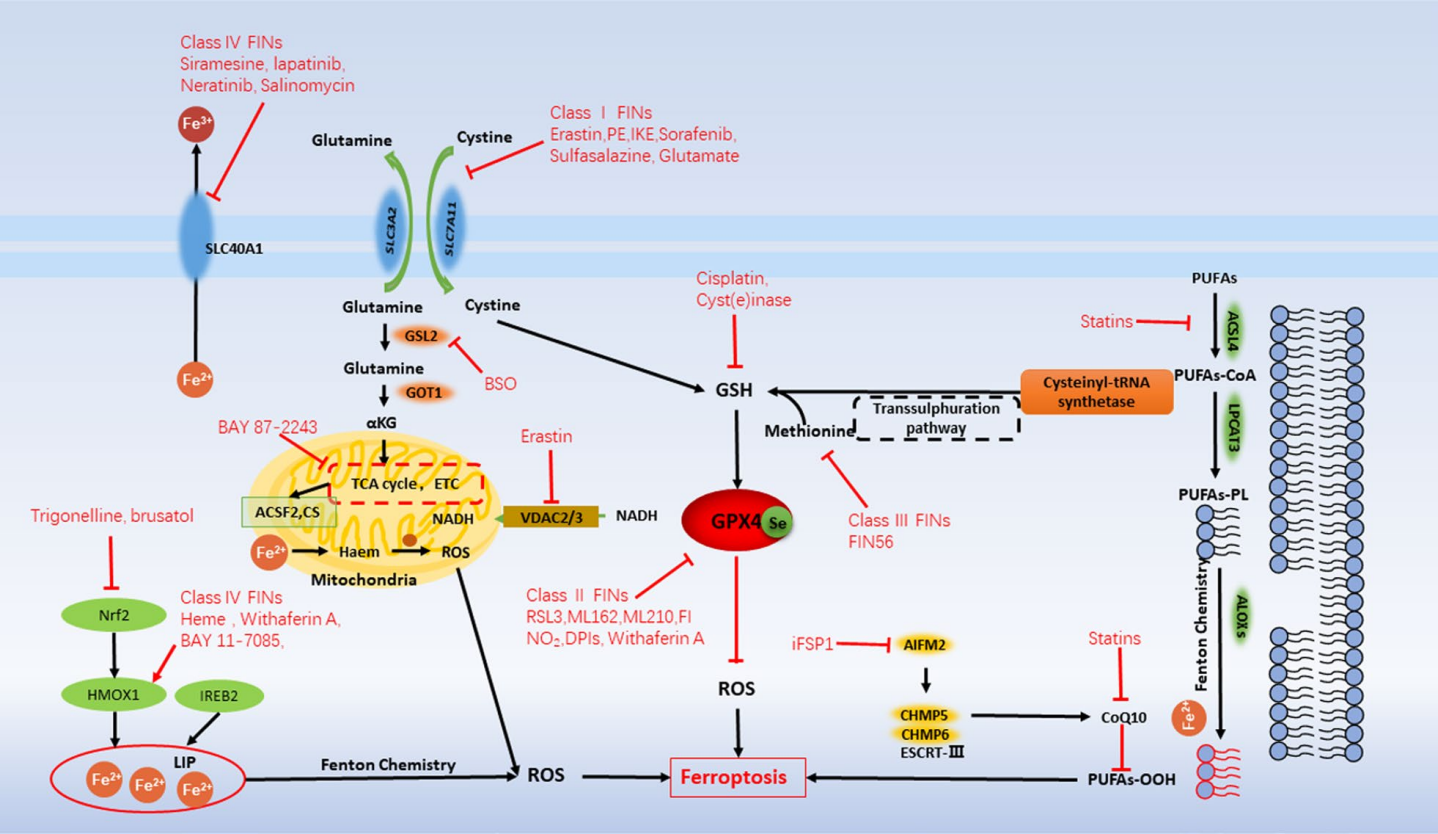
The inducers of ferroptosis

The concentration of GSH in cancer cells is more than 1000 times that of extracellular cells and 4 times that of normal cells. GSH plays an important role in scavenging superoxide and resisting cell death [7, 91]. So GSH has been considered as cancer’s Achilles’ heel [51] (Fig. 4).

SystemXc- and the transsulfur pathway are the two main sources of cysteine and GSH synthesis. In some cancers, apparent silencing or loss of transsulfase enzymes makes cells more dependent on SLC7A11 regulated cystine uptake pathways [91, 92]. After suppressing the System Xc-, SLC7A11 will be compensated up. These changes can be used as pharmacodynamic biomarkers to identify SLC7A11 inhibition and ferroptosis [65]. Based on this, SLC7A11 is considered a very good anti-cancer drug target.

Among Class I FINs, erastin is the prototype of ferroptosis inducer, which can reduce GSH level by directly inhibiting system Xc-, and has been found to inhibit the growth of cervical cancer and ovarian cancer cells [93]. In addition to system Xc-, erastin also targets mitochondrial voltage-dependent anion channel (VDAC) and RAS genes. Knockdown or silencing of these genes will trigger cell resistance to erastin [30]. In addition to causing ferroptosis in cancer treatment, erastin has also been shown to enhance the chemotherapy effects of traditional anti-cancer drugs (such as doxorubicin, cisplatin, temozolomide, cytarabine, etc.) in certain cancer cell lines [94–96]. Although erastin has an inhibitory effect, its poor water solubility and unstable metabolism limit its application in the body. Therefore, scientists have developed erastin derivatives piperazine erastin (PE) and imidazolone erastin [28], which act on cancer cells in a similar manner to erastin. However, PE and IKE show better water solubility and stability than their prototypes in the physiological environment, and effectively inhibit tumor growth in experimental models of fibrosarcoma and diffuse large B cell lymphoma (DLBCL) [33, 97]**.**

In addition, the FDA-approved immunosuppressant sulfasalazine (SAS, trade name Azulfidine, Salazopyrin, Sulazine, etc.) is used as a first-line treatment for rheumatoid arthritis and can also be used as a System Xc- inhibitor. The SAS mode of action is similar to erastins, but the effect is much weaker. It is currently used to treat lymphoma, pancreatic cancer and lung cancer [98–100]. However, due to its poor pharmacokinetics, lower potency and metabolic stability, the clinical application of this drug is limited [101, 102]. SAS is also used as a combination therapy to enhance the therapeutic effect of other chemotherapy on glioma [103]. Sorafenib is an anti-cancer drug approved by the FDA for the treatment of HCC, RCC and thyroid cancer. In addition to inhibiting receptor tyrosine kinases, it can also promote cell ferroptosis by inhibiting the function of System Xc-. Sorafenib was determined to be class I FINs [51]**.**

BSO is an ferroptosis inducer that directly blocked the synthesis of GSH, which can inhibit the growth of mouse breast cancers and increase the melphalan chemosensitivity of melanoma and neuroblastoma cells [104–106]. With the deepening of research, other inhibitors that inhibit GSH synthesis have also been discovered and verified in different cancer cells. Finally, as a genetic approach to increase the efficacy of GSH depletion, an optimized human cystathionine glyase (CGL), coined cyst(e)inase, was engineered to degrade cysteine and cystine with a higher kinetic rate. This approach impedes the growth of prostate and breast cancer xenografts and increases mouse survival in a chronic lymphocytic leukemia model [51, 107]**.**

### Class II and III FINs

Class I FINs are a promising anti-cancer drug for cells with high SLC7A11 expression. However, in other cancer cells, there may be other ways to resist ferroptosis caused by class I FINs. For example, the up-regulation of the heat shock protein (HSPB1) can make cells resistant to the treatment of class I FINs [98]. If inhibiting the synthesis of GSH fails to induce ferroptosis in some cells, it may be due to the existence of the transsulfur pathway that the cells can still survive [105, 108] (Fig. 4). Therefore, for this type of cancer cells, the targeted inactivation of the activity of GPX4 by class II and III FINs can induce ferroptosis of the cells. At present, among many class II FINs, RSL3 can induce ferroptosis by directly targeting GPX4. RSL3 targets enzymes with nucleophilic sites, and directly inactivates GPX4 through the alkylation of selenocysteine [33]. At present, RSL3 is widely used. For example, in mouse models, it can block the activity of GPX4 to promote ferroptosis and thus inhibit the growth of fibrosarcoma [33]. Withaferin A inhibits the growth and recurrence rate of neuroblastoma xenografts [82]. Altretamine is an FDA-approved anti-cancer drug for the treatment of ovarian cancer, which induces ferroptosis through GPX4 inhibition [77]. In class III FINs, the latest iFSP1 small molecule inhibitors developed by American and German scientists can block the function of the ferroptosis inhibitor the protein FSP1 and reduce the content of ubiquinone to increase lipid peroxidation [109, 110]. FIN56 is an ferroptosis inducer derived from CIL56, which was discovered through regulatory analysis of 56 lethal compounds that do not depend on caspase. FIN56 leads to depletion of GPX4 and CoQ10 through the SQS-mevalonate pathway [111]**.**

### Class IV FINs

Compared with normal cells, cancer cells have a stronger demand for iron, which makes cancer cells more sensitive to the induction of ferroptosis. Based on the characteristics of cancer cells, class IV FINs mainly promote the synthesis of lipid peroxides by increasing intracellular LIP or iron oxide to induce ferroptosis, and may provide new opportunities for cancer treatment [112]. In addition to make GPX4 inactivation, withaferin A also increases LIP through HMOX1-mediated heme degradation, thereby inducing ferroptosis in neuroblastoma [82]. Heme and ferrous ammonium sulfate induce intracellular iron accumulation, which leads to ferroptosis of neuroblastoma cells [82]. BAY87-2243, a known inhibitor of IκBα, can up-regulate the expression of HMOX1 and enrichment of iron ions in an NFκB-dependent manner, and induce ferroptosis [83]. FINO_2_ is a class of organic peroxides, which share many characteristics with artemisinin (for example, iron is required to induce cell death, and ROS is produced). The peroxide containing 1,2-dioxolane structure has been identified as ferroptosis inducers, which are more effective than artemisinins in some cancer cell lines. The ferroptosis caused by FINO_2_ is due to the combined effects of the direct oxidation of unstable iron and the inactivation of GPX4 (Fig. 4). Compared with non-malignant cells in the same tissue, FINO_2_ is more effective in malignant cells. In addition, in vitro experiments have shown that FINO_2_ can bypass chemoresistance-related pathways (eg., p53 mutations, BCL-2 overexpression) [55]. The combination of the kinase tyrosine inhibitor lapatinib and the lysosomal drug silamexine that destabilizes the lysosomes synergistically induces ferroptosis by disrupting iron transport in breast cancer cells [113]. The use of iron oxide nanoparticles to promote iron overload has been shown to induce ferroptosis and inhibit cancer growth in nutrient-deficient cancer cells [114]. In addition, recently FDA approved polyethylene glycol-coated ultra-small nanoparticles Coined Cornel dots(C'dots) can absorb and integrate extracellular iron and transport it into the cell, causing iron overload and inducing cell death [114]**.**

## Ferroptosis and cancer therapy

Although breakthroughs have been made in the field of cancer therapy, cancers are still the second leading cause of death in the world. At present, the main treatment approach is to use anti-cancer drugs to trigger the apoptotic death of cancer cells. However, due to the inherent and acquired resistance of cancer cells to apoptosis, the therapeutic effect is limited. Drug resistance is still the main limiting factor for the cure of cancer patients. Inducing ferroptosis of cancer cells is one of the best ways to avoid drug resistance [51, 131]. It can be through the use of exogenous molecules or drugs, or regulation of extracellular physiological conditions (eg., high concentration of extracellular glutamate) blocking system xCT to induce ferroptosis extensively, and it can also target cytogenesis according to the difference between cancer cells and normal cells. At present, several small molecules and FDA-approved clinical drugs activate ferroptosis in cancer cells, and the efficacy of ferroptosis inducers to inhibit cancers in various experimental models. At the same time, there are also a variety of treatments that can be effectively induced ferroptosis in experiments, emphasizing the potential as a new type of anti-cancer therapy [51, 129, 131]. Next, we will introduce how some current treatments use ferroptosis to treat cancer (Table 2).

**Table 2:** Ferroptosis and cancer therapy

Therapy	Treatment	Combination drugs	Mechanism	Tumour type	Refs
Chemotherapy	Sorafenib	siRNA	Inhibit the MT-1G and the system xc-	HCC, RCC, NSCLC, PDAC	[34, 67, 134, 135]
	Artemisinin	Iron	Increase the level of intracellular free iron	PDAC, AML, HNSCC	[122–125]
	Cyst(e)inase	FINs	Deplete extracellular cystine	PDAC, Prostate cancer, Chronic lymphocytic leukemia	[109, 116]
	Statins	-	Reduce selenoproteins (such as GPX4) and CoQ10 biosynthesis	Breast Cancer	[138]
Radiotherapy	RT	FINs	Up-regulates ACSL4, inhibit SLC7A11 or GPX4	Neuroblastoma, NSCLC, Fibrosarcoma, Melanoma, Breast Cancer, Cervical cancer	[140–145]
Immunotherapy	PD-L1 inhibitors	FINs	Releas IFNγ to reduce the uptake of cystine	Fibrosarcoma	[11]
	TGF-β inhibitors and PD-1 antibodies	FINs	Generate an immunogenic microenvironment and produce H_2_O2, promoting the Fenton reaction	Melanoma	[146]
Nanomedicine	Metal–Organic Frameworks (MOF)	–	Release iron	Mononuclear macrophage leukemia	[148]
	FePt-NP2	Iron nanoparticles and cisplatin	Increase the sensitivity of cancer cells to cisplatin	Ovarian cancer	[117]
	SRF@FeIIITA (SFT)	Fe^3+^ and TA, nanocrystals of SRF,	Inhibit GPX4 and generate ROS	Fibrosarcoma	[71]
	Nano-delivery vehicle	WithaferinA, IKE	Improve solubility and biocompatibility, and increase accumulation	Neuroblastoma, DLBCL	[84, 99]
		AMSNs	Target cancer by ASS and release Mn ion to consume GSH	HCC	[149]
		MON-p53	Providie unstable iron, and deliver p53 to cells	Fibrosarcoma	[150]
	Ultra-small poly(ethylene glycol) coated silica nanoparticles		Transport of extracellular iron into the cell	NeuroblastomaHCC	[151]
	PDT	FINs	Produce ROS and consume O_2_	OTSCC, Breast Cancer, HCC	[152–157]
	PTT	SRF@MPDA-SPIO, FPMF@CpGODN	Release iron and SRF, produce ROS and consume O_2_	CRC, Breast Cancer	[121, 158, 159]

*HCC* hepatocellular carcinoma, *RCC* renal cell carcinoma, *PDAC* pancreatic ductal adenocarcinoma, *HNSCC *head and neck squamous cell carcinoma, *NSCLC* non- small- cell lung cancer, *AML* acute myeloid leukaemia, *DLBCL *diffuse large B cell lymphoma, *GSH* glutathione, *TA *tannic acid, *SRF *sorafenib, *ASS *arginine succinate synthase, *PDT *photodynamic therapy, *PTT* photothermal therapy

### Ferroptosis and chemotherapy

Chemotherapy is one of the main treatment methods for malignant cancers, but in the course of cancer chemotherapy, various mechanisms have led to cancer multidurg resistance (MDR), and resistance to chemotherapy drugs has become the main reason of chemotherapy failure in cancer patients [132]. In recent years, more and more studies have been conducted on how to effectively overcome cancer MDR. With the ferroptosis into the eyes of researchers, there is hope for overcoming cancer chemotherapy resistance [132, 133]. It is currently known that inhibiting xCT and GPX4 can effectively enhance the sensitivity of tumors (eg., pancreatic ductal carcinoma, NSCLC and osteosarcoma) to gemcitabine and cisplatin [118, 119]. In addition, there are several other drugs that are already in clinical use or have strong clinical transformation potential that can promote ferroptosis.

#### Sorafenib

Sorafenib is a clinically approved multi-kinase inhibitor for the treatment of advanced cancers [65]. Studies have shown that in the treatment of HCC, RCC, lung cancer or pancreatic cancer, the anti-cancer activity of sorafenib mainly depends on inducing ferroptosis by inhibiting the activity of the system xc-, rather than relying on the inhibition of its kinase [34, 65, 134]. However, in certain cancer cell lines, drug resistance has been observed in sorafenib-mediated cancer therapy. In the study of drug-resistant cancer cells, it is found that the target gene of metallothioneins-1G (MT-1G) is a biomarker and contributing factor of sorafenib resistance [34, 135]. Therefore, inhibiting the MT-1G pathway during sorafenib treatment can reduce the risk of chemotherapy resistance and improve the therapeutic effect [130]**.**

#### Artemisinin

In addition to its therapeutic value in the treatment of malaria, artemisinin also has a killing effect on a variety of cancers. In addition to inducing cell apoptosis, artemisinin (especially artesunate and dihydroartemisinin) can also increase the level of intracellular free iron by promoting ferritin autophagy, thereby triggering ferroptosis in cancer cells [122–125]. Iron supplements, such as holotransferrin, can enhance the anti-cancer properties of artemisinin [136]. This is because cancer cells have more heme, which favors the cancer targeting specificity of artemisinins in a similar manner as in the case of malaria [137]. In clinical trials, artemisinin has been proved to be effective in treating acute myeloid leukemia, [123] and head and neck squamous cell carcinoma (HNSCC) [122]**.**

#### Cyst(e)inase

Cyst(e)inase is an engineered human enzyme that can effectively degrade cysteine and cystine (cyst(e)ine) in serum. Subsequent depletion of extracellular cystine leads to the death of prostate cancer and chronic lymphocytic leukemia cells in vitro and in vivo [107]. Cyst(e)inase-mediated depletion of cyst(e)ine can induce ferroptosis in pancreatic without causing obvious toxicities, suggesting acceptable safety and tolerability [116]. The strategy of using cystinase to regulate extracellular cystine levels can provide new therapeutic opportunities for ferroptosis-based anticancer therapies, especially with drugs that induce ROS (eg., doxorubicin, gemcitabine, paclitaxel, 5-fluorouracil, Bortezomib).

#### Statins

Statins (eg., fluvastatin, lovastatin and simvastatin) are a class of drugs used to hypotensive by inhibiting HMG-CoA reductase (HMGCR). By inhibiting the mevalonate pathway, statins can reduce selenoproteins (such as GPX4) and CoQ10 biosynthesis, thereby enhancing ferroptosis [111, 129]. Data from clinical trials indicate that atorvastatin and fluvastatin may have anti-proliferative effects in cancers overexpressing HMGCR [138, 139]. A deeper understanding of the ferroptosis pathway regulated by cholesterol may help to better use statins in future clinical studies (Fig. 3).

### Ferroptosis and radiotherapy

Radiotherapy (RT), as one of the effective cancer treatments, uses ionizing radiation (IR) from a radioactive source to cause DNA damage and cause cell apoptosis. And now studies have found that RT can directly induce ferroptosis of cancer cells [120]. Ataxia telangiectasia-mutated (ATM) is a key protein kinase in the process of DNA damage repair. The ATM-mediated down-regulation of SLC7A11 caused by RT is the cause of ferroptosis in cancer cells [140]. When the SLC7A11 is overexpressed, it can promote RT resistance by inhibiting ferroptosis [126]. Studies have shown that FINs (such as RSL3, erastin, sorafenib, and sulfasalazine) can synergistically enhance RT efficacy by inhibiting SLC7A11 or inactivating GPX4 in models of glioma, lung cancer, fibrosarcoma, melanoma, breast cancer, and cervical cancer [60, 120, 126, 140–145]. In addition to down-regulating SLC7A11, RT also up-regulates ACSL4, thereby increasing lipid synthesis and peroxidation, inducing ferroptosis [126]**.**

These studies have revealed the molecular mechanism between ferroptosis and RT sensitization, provide a theoretical basis for further elucidating the mechanism of ferroptosis in RT sensitization, and have groundbreaking significance for the development of ferroptosis-related drugs with RT sensitization.

### Ferroptosis and immunotherapy

**Fig. 5:**
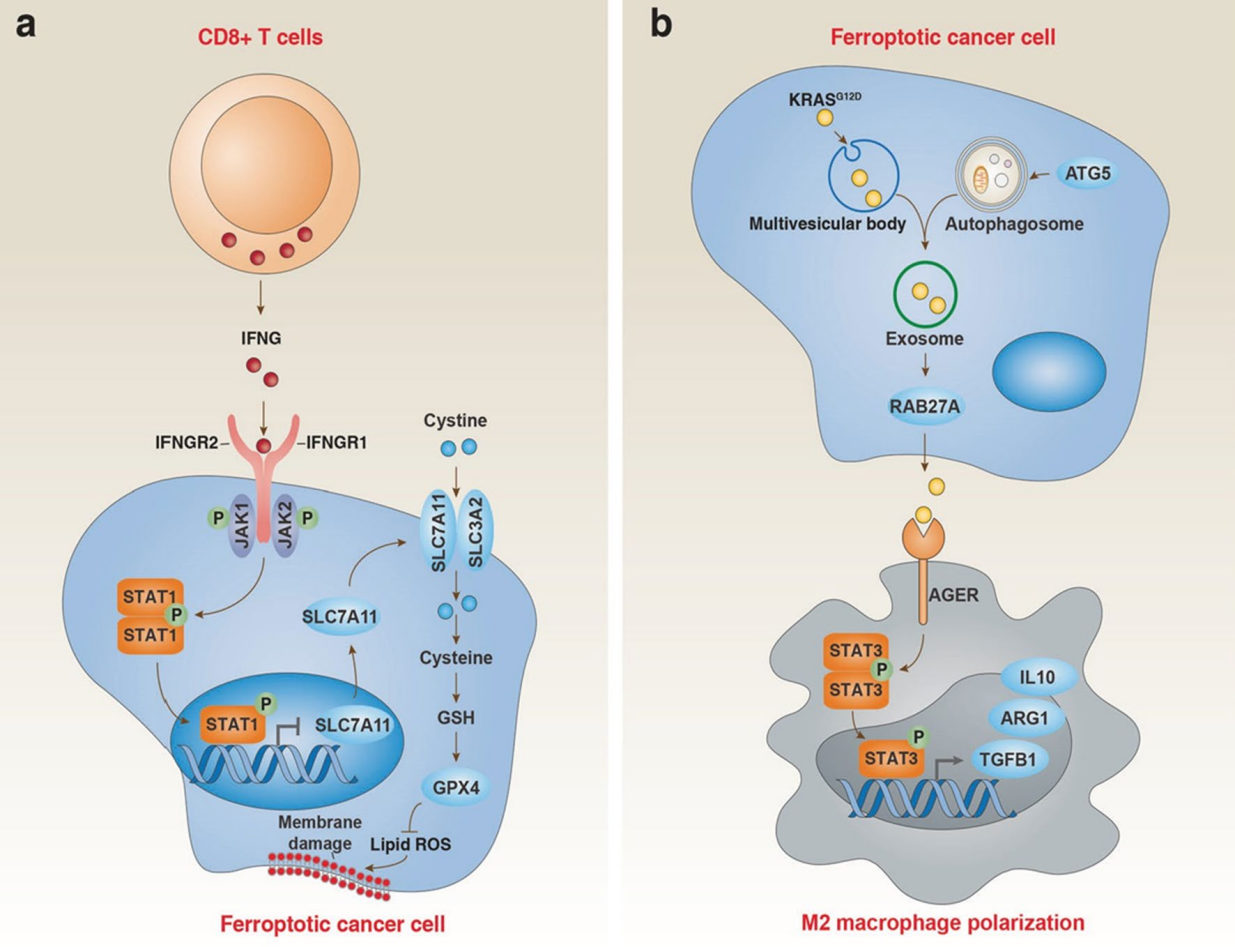
Dual role of ferroptosis in tumor immunity. **a** CD8^+^ T cell-mediated IFNG release inhibits SLC7A11 expression in cancer cells through activation of the STAT1 pathway, thereby inducing tumor cell ferroptosis. **b** Ferroptotic cancer cell-mediated KRAS^G12D^ release increases M2 macrophage polarization through activation of the STAT3 pathway, thereby limiting antitumor immunity

Immunotherapy is currently one of the promising treatment methods for anti-cancer. It is achieved by activating the immune system and enhancing its inherent cancer treatment capabilities. Immune checkpoint inhibitors (ICIs) mainly act by activating effective anti-cancer immune responses driven by cytotoxic T cells. The currently approved ICIs target CTLA4, PD-1 and their ligand PD-L1. Judging from ongoing clinical trials, PD-1/L1 inhibitors are mainly be used for combination therapy, with targeted therapy or other immunotherapy. In recent years, it has been found that ferroptosis is closely related to immune regulation. For example, PD-L1 antibodies can promote lipid peroxidation-dependent ferroptosis in cancer cells, PD-L1 antibodies and ferroptosis inducers synergistically inhibit tumor growth in vitro and in vivo [11]. Cytotoxic T cell-driven immunity can induce ferroptosis in cancer cells. In terms of mechanism, CD8 + T cells can down-regulate the expression of SLC3A2 and SLC7A11 by releasing IFNγ, thereby reducing the uptake of cystine and promoting lipid peroxidation in cancer cells [11]. TGFβ1 can promote ferroptosis through SLC7A11 transcription inhibition and ZEB1 activation. However, TGF-β inhibitors and PD-1 antibodies can synergistically generate an immunogenic microenvironment and produce H_2_O_2_, thereby promoting the Fenton reaction, triggering ferroptosis of cancer cells. And the cancer antigens released after cell death in turn promote the immunogenicity of the microenvironment [146]. Under certain circumstances, ferroptosis may have a cancer-promoting effect. The release of damage-related molecular patterns (DAMP) in exosomes during death, which ultimately leads to the polarization of macrophages to the M2 phenotype and stimulates tumor growth [147]. These findings bridge the gap between ferroptosis and immunotherapy, laying a theoretical foundation for the synergy of ferroptosis-immunotherapy in the treatment of malignant cancers. At the same time, it provides a direction for further exploring the molecular mechanism of ferroptosis to promote the efficacy of immunotherapy (Fig. 5).

### Ferroptosis and nanomedicine

The current clinical treatments are far from satisfactory due to various reasons. Combining emerging biological discoveries and traditional treatment methods has become the development trend of effective cancer treatment. There have been studies on ferroptosis-related nano-preparations combined with other treatment methods to enhance the therapeutic effect of cancers. One of the anti-cancer strategies is to use nano-drugs to induce ferroptosis.

The most direct method is to develop iron-containing nanoparticles, which can transport and release iron into cells. At present, in addition to solid iron-based nanocrystals, some other iron-based nanocomposites have been developed, such as amorphous iron (Fe0) nano-metallic glass and metal organic framework (MOF) have been developed to effectively release iron in TME, improving the iron release efficiency [148]. Iron nanoparticles kill cancer cells by increasing iron levels and ROS. At the same time, chemotherapeutic drugs, such as cisplatin prodrugs, can also be modified on iron nanoparticles to form FePt-NP2 to increase the sensitivity of cancer cells to cisplatin [117]. Or add iron to the nanoparticles of chemotherapy drugs, such as depositing Fe^3+^ and tannic acid (TA) on the nanocrystals of SRF, resulting SRF@FeIIITA (SFT). After entering the cell, it can be destroyed in the lysosomal acidic microenvironment, thereby releasing SRF to inhibit GPX4. At the same time, TA is an acid-activated reducing agent that can reduce Fe^3+^ to Fe^2+^ to generate ROS and enhance ferroptosis [69]. In addition, methylene blue (MB) is loaded into this SFT nanoplatform for bioimaging guided photodynamic therapy (PDT) [69]**.**

Another intuitive method is to add FINs to the nano-delivery vehicle. Compared with free drugs, the nano-platform can improve solubility and biocompatibility, and increase accumulation through active or passive targeting, improve the pharmacokinetics of FINs, and improve the therapeutic effect [97]. For example, nanoparticle formulations of withaferin A promoted accumulation due to increased permeability and EPR retention effects and inhibited tumor growth in neuroblastoma [82, 102]. Similarly, the use of IKE nanoparticles resulted in increased accumulation of IKE in DLBCLs, suppressing tumor growth in mice while reducing toxicity [97]**.**

In addition to small molecule drugs, nanocarriers can also be combined with other small molecules, such as amino acids, plasmids and other unconventional drugs. Some researchers have developed arginine-terminated manganese silicate nanobubbles (AMSNs) [149]. Due to the deficiency of arginine succinate synthase (ASS) in cancer, AMSN has high biocompatibility and strongly cancer targeting ability. After entering cells, AMSN releases high-valence manganese ions (Mn^3+^ and Mn^4+^), which are reduced to Mn^2+^ by GSH. The consumption of GSH leads to increased intracellular oxidative stress and inactivation of GPX4. In addition, the magnetic hollow structure enables AMSN to be used as a bioimaging contrast agent and drug delivery carrier [149]. Other researchers have also developed a metal–organic network (MON-p53) encapsulated by p53 plasmids, which can kill cancer cells through a mixed ferroptosis/apoptosis pathway, in which ferroptosis plays a major role. MON-p53 nanoparticles induce ferroptosis by providing unstable iron, and also deliver p53 to cancer cells for gene therapy, effectively inhibiting cancer growth and metastasis [150]**.**

What's more attractive is that nanomaterials can not only serve as carriers, but they can also induce ferroptosis by participating in biochemical reactions and disturbing metabolic balance. In 2016, the first nanoparticle ferroptosis inducer was reported [114]. The FDA-approved ultra-small poly(ethylene glycol) coated silica nanoparticles create Cornel dots (C′ dots), which induce ferroptosis in amino acid starved cancer cells and inhibit cancer growth. Researchers found that the PEGylated C′ dots absorbs extracellular iron and binds to it. When it enters cancer cells, it releases it and increases intracellular iron levels. Accompanied by the production of ROS and the consumption of GSH, it eventually leads to ferroptosis [151]**.**

Researchers have used the tunable physicochemical properties of nanomaterials to study the combined treatment effects of ferroptosis and phototherapy in addition to the classic treatment. These studies mainly focus on the aspects of photodynamic therapy (PDT) and photothermal therapy (PTT), because they are intrinsically related to ROS and O_2_, and ferroptosis can produce ROS and provide O_2_ [152, 153]. The researchers prepared a nanosystem assembled by the photosensitizer Ce6 and erastin. The results showed that the Ce6-erastin nanoassembly produced a large amount of toxic reactive oxygen species under localized cancer irradiation, and showed strong anti-cancer activity on xenograft cancers [154]. Studies have also found that the ferroptosis inducer SRF has a synergistic effect with PDT, and the ferroptosis inhibitor DFO can reverse the anti-cancer ability of PDT [155–157]. Recently, scientists loaded SRF and SPIO nanoparticles on the mesopores and surface of MPDANPs to form SRF@MPDA-SPIO, which can release Fe^3+^/ Fe^2+^ and SRF in cells, which can be used for MR imaging-guided ferroptosis—PTT combination therapy [158]. In addition, there is a high-efficiency nanosystem that combines chemotherapy, ferroptosis and PTT triple therapy for the combined treatment of ER + breast cancer [121]. Other researchers have constructed the nanocomposite FPMF@CpGODN, which is a perfect combination of ferroptosis, PTT, chemotherapeutics and immunotherapy, which has a stronger effect on eliminating primary tumors and preventing tumor recurrence [159]. This concept of combining ferroptosis with nanomaterials, chemotherapeutics, immunotherapy and other treatment methods can provide a new clinical vision for cancer treatment.

Although the existing nanomaterials have produced good ferroptosis effects, there are still many problems to be solved before entering the clinic. The current biosafety of nanomaterials still needs to be improved in many ways, such as biological targeting, biocompatibility, biodegradability and immunogenicity. At the same time, we must continue to develop new types of nanomaterials that can induce ferroptosis and suitable for making the nanomedicines. In general, although nanomaterials can induce ferroptosis of tumor cells and can achieve ideal tumor suppression effects, almost all research data comes from experimental animals, so there are still many preclinical experiments that need to be further carried out.

## Conclusions and perspectives

Although much progress has been made in tumor biology and therapeutics, there is still a long way to go to win the fight against cancer. Since ferroptosis was discovered, researchers in the field of biomedicine have been enthusiastic about it. How to identify ferroptosis, how to regulate ferroptosis, how to induce ferroptosis, and how to apply ferroptosis has gradually become a research hotspot among researchers. As the above problems are solved one by one, ferroptosis has received more and more attention in tumor biology and tumor treatment. In various cell or animal cancer models, ferroptosis has a significant anti-cancer effect. Targeted regulation of ferroptosis in tumor cells has become an emerging anti-cancer strategy. Further exploration of unknown key molecules or pathways of ferroptosis can provide new targets and new methods for tumor treatment. For example, the induction of ferroptosis through nanomedicine can not only enrich tumor treatment methods, but also treat malignant tumors in combination with traditional treatment methods. There are still many unknowns and challenges in the basic research and clinical transformation of ferroptosis. However, we believe that with the deepening of basic research on ferroptosis, future research results on induction of ferroptosis can provide more theoretical guidance and programs for tumor treatment.

## Data Availability

The data sets supporting the results of this article are included within the article.
